# Chromosome-Level Genome Assembly of *Protosalanx chinensis* and Response to Air Exposure Stress

**DOI:** 10.3390/biology12091266

**Published:** 2023-09-21

**Authors:** Yanfeng Zhou, Xizhao Zhang, Xuemei Tang, Yifan Zhou, Yuting Ding, Hong Liu

**Affiliations:** 1 College of Fisheries, Key Lab of Freshwater Animal Breeding, Ministry of Agriculture and Rural Affair, Engineering Research Center of Green Development for Conventional Aquatic Biological Industry in the Yangtze River Economic Belt, Ministry of Education, Huazhong Agricultural University, Wuhan 430070, China; 2 Key Laboratory of Freshwater Fisheries and Germplasm Resources Utilization, Ministry of Agriculture and Rural Affairs, Freshwater Fisheries Research Center, Chinese Academy of Fishery Sciences, Wuxi 214081, China; zhangxizhao@ffrc.cn; 3 Wuxi Fisheries College, Nanjing Agricultural University, Wuxi 214081, China

**Keywords:** *Protosalanx chinensis*, chromosome-level genome, air exposure stress

## Abstract

**Simple Summary:**

In the present study, we assembled a high-quality chromosome-level genome of *Protosalanx chinensis*, which is the first chromosome-level genome for Salangidae. These genomic data provide a fundamental resource for ecological and adaptation studies of *Protosalanx chinensis*, and offer a deeper understanding of the response to air exposure stress and species conservation.

**Abstract:**

*Protosalanx chinensis* is a suitable particular species for genetic studies on nearly scaleless skin, transparency and high sensitivity to hypoxia stress. Here, we generated a high-quality chromosome-level de novo assembly of *P. chinensis*. The final de novo assembly yielded 379.47 Mb with 28 pseudo-chromosomes and a scaffold N50 length of 14.52 Mb. In total, 21,074 protein-coding genes were predicted. *P. chinensis*, *Esox lucius* and *Hypomesus transpacificus* had formed a clade, which diverged about 115.5 million years ago. In the air exposure stress experiment, we found that some genes play an essential role during *P. chinensis* hypoxia, such as *bhlh*, *Cry1*, *Clock*, *Arntl* and *Rorb* in the circadian rhythm pathway. These genomic data offer a crucial foundation for *P. chinensis* ecology and adaptation studies, as well as a deeper understanding of the response to air exposure stress.

## 1. Introduction

**Figure 1:**
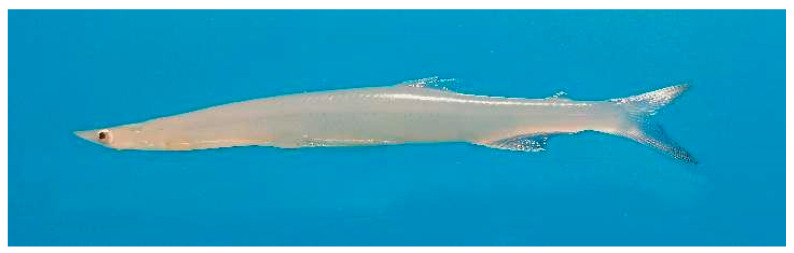
Illustration of *P. chinensis*.

*Protosalanx chinensis* (family Salangidae, order Salmoniformes, Figure 1) is a small annual cold-temperature fish endemic to East Asia and has some specific morphological characteristics, including transparency and scaleless skin [1,2,3,4]. *P. chinensis* exhibits strong ecological plasticity, with populations found in both freshwater and seawater habitats, including the Yangtze River Basin and its associated lakes (Taihu Lake, Hongze Lake, etc.), as well as the offshore waters of the Yellow Sea, Bohai Sea and East China Sea [1,5,6]. Due to the important economic value of *P. chinensis*, it was widely translocated to many lakes and reservoirs in northern China in the mid-1980s, and gradually formed a stable population [7]. This deliberate act of artificial translocation has engendered a remarkable shift, resulting in a notable proliferation of *P. chinensis* across diverse aquatic habitats, effectively amplifying its presence [7]. Studies of the genomic and physiological characteristics of *P. chinensis* have helped us to better understand the environmental adaptations of *P. chinensis*.

*P. chinensis* is a valuable model for studying the molecular mechanisms underlying the evolution of hypoxia. *P. chinensis* is difficult to obtain alive and can die quickly when stressed by hypoxia during net fishing [1]. Previous studies on the genome of *P. chinensis* have focused only on phylogeny, sexual differentiation, and skeletal development [2,3]. A basic understanding of stress and the corresponding physiological state of *P. chinensis* is still lacking. An air exposure stress experiment is an effective experimental tool with which to understand the stress state. An air exposure experiment on gilthead seabream showed that stress-induced hormonal changes affected the liver’s metabolic organization and highlighted the crucial role of vasotocinergic and isotocinergic pathway [8]. A study of rainbow trout demonstrated changes in miRNAs in fish blood during air exposure and identified several miRNA markers [9]. As an economically valuable fish, *P. chinensis* has not yet been fully cultured in captivity and transported live, so there is potential value in studying its response to hypoxic stress.

With the development of genomic (particularly long-reads) sequencing, two draft genome assemblies of *P. chinensis* had recently been reported with assembly quality with a contig N50 of 17.2 Kb [2], and a contig N50 of 103 Kb [3], respectively (*Protosalanx hyalocranius* and *Protosalanx chinensis* were the same species). Although these two genome drafts provide preliminary genetic information of *P. chinensis*, these genomes are deficient due to the limitation of sequencing technology. Therefore, chromosome-level genomes and comparative genomics resource are essential to understanding ecological and evolutionary research, translocation adaptation, and genetic improvement.

Here, we generated a high-quality chromosome-level de novo assembly of *P. chinensis*. A set of protein-coding genes was annotated, and the evolutionary history of *P. chinensis* was analyzed. In the air exposure stress experiment, the expression pattern of differentially expressed genes (DEGs) was investigated. These genomic data offer a crucial foundation for *P. chinensis* ecology and adaptation studies, as well as a deeper understanding of the response to air exposure stress and species conservation.

## 2. Materials and Methods

### 2.1. Sample Collection and DNA and RNA Sequencing

We collected muscle samples from an adult *P. chinensis* individual in the Hongze Lake at Jiangsu, China, for sequencing (Figure 1). After the muscle samples were collected, they were rapidly frozen in liquid nitrogen and stored at −80 °C until DNA extraction. DNA was extracted from muscle tissue. RNA was extracted from the larvae. DNA was extracted following the phenol/chloroform DNA extraction method. After the extraction of the DNA and RNA, corresponding quality control was conducted according to different library construction types. The quality control included assessing the concentration, purity, and fragment integrity of the samples.

With the BGI MGISEQ platform, a short insert WGS library was generated according to the manufacturer’s recommendations. A PacBio HiFi library was constructed using a QIAGEN Blood & Cell Culture DNA Midi Kit following the manufacturer’s instructions (QIAGEN, Hilden, Germany) and then sequenced on the PacBio Sequel II system. A Hi-C library was generated using the *Mbo* I restriction enzyme and sequenced on the BGI MGISEQ platform. We constructed one PacBio HiFi library with an insert fragment size of approximately 15 kb, and one Hi-C library with an insert fragment size of approximately 300 bp.

Fifteen RNA libraries were constructed using the TRIzol Total RNA Isolation Kit (Takara, San Jose, CA, USA) after which the concentration and purity of the extracted RNA were measured to ensure quality. Subsequently, the RNA was fragmented into appropriate lengths using digestion enzymes. This fragmented RNA was then reverse-transcribed to synthesize cDNA, which underwent end repair, addition of specific adapter sequences, and PCR amplification for library construction. Finally, the constructed RNA library was sequenced on the BGI MGISEQ platform.

### 2.2. Sequencing QC and Genome Assembly

We used the SOAPnuke v2.1.7 [10] pipeline to filter out the low-quality and adaptor reads. After that, we calculated the K-mer (*k* = 21) frequency distribution with Jellyfish v2.2.6 [11] and analyzed the result using GenomeScope v1.0 [12]. HiFi reads with about 62× coverage were sequenced using the PacBio Sequel platform and cleaned with SMRTLink v8. The contig assembly was carried out using Hifiasm v0.16.1-r375 [13], followed by a removal of the redundant sequences with the Purge-Haplotigs [14] program. Subsequently, the contigs were further connected to the chromosome level using the Juicer v1.5 [15] and 3D-DNA v180922 [16] pipelines. The BUSCO completeness score of the *P. chinensis* genome was calculated using BUSCO v5.2.2 [17] based on the actinopterygii (odb10) dataset.

### 2.3. Identification of Repetitive Sequences

We identified the repetitive sequences using a combination of de novo and homolog-based methods. For de novo annotation, we used RepeatModeler v1.0.4 (http://www.repeatmasker.org/RepeatModeler/, accessed date: 23 February 2023) and LTR-FINDER v1.0.7 [18] software to construct a primary library. This customed library was used to screen repeat sequences via the program RepeatMasker v4.0.7 [19]. For the homolog-based prediction, we utilized RepeatMasker v4.0.7 [19], RepeatProteinMasker v4.0.7 [19] and Tandem Repeat Finder v4.10.0 [20] based on the Repbase database.

### 2.4. Genome Annotation

Gene prediction was conducted through a combination of homology-based prediction, ab initio prediction and transcriptome-based prediction methods. Next, 96.4 Gb RNA-seq data were directly mapped to *P. chinensis* assembly with Hisat2 v2.1.0 [21] to identify putative exon regions and splice junctions. StringTie v1.3.5 [22] was then used to assemble the mapped reads into gene models and validated using PASA v2.5.2 [23]. Finally, we identified the candidate coding regions by employing TransDecoder v5.5.0 (https://github.com/TransDecoder/TransDecoder, accessed date: 23 February 2023). For homology-based annotation, we downloaded the assemblies and gene annotation files of four actinopterygii species (*Danio rerio*, *Oryzias. latipes*, *P. hyalocranius* and *Salmo salar*) from the NCBI database. Combined with the above RNA-seq and homolog data, we predicted the homology-like coding sequences using GeMoMa v1.8 [24]. A total of 1200 high-quality coding genes were used to train the predictors using August v3.2.1 [25] and SNAP v2006-07-28 [26] (Korf, 2004) and then ab initio prediction was performed. Lastly, we integrated all the protein-coding genes predicted using the above three strategies with the EVidenceModeler (EVM) pipeline v1.1.1 [23].

### 2.5. Phylogenetic and Gene Family Analysis

We used OrthoFinder v2.3.11 [27] to cluster protein-coding genes. Single-copy orthologous genes (1:1:1) were aligned using MAFFT v7.310 [28]. Referring to the methods used in previous studies on *P. chinensis* [3], we used PhyML v3.3 [29] with the HKY85 model to construct a maximum-likelihood phylogenetic tree with 100 pseudoreplicates. All branches had 100/100 bootstrap support, showing phylogeny consistent with a previous study [3]. We estimated the species divergence time using MCMCTREE in PAML v4.9 [30]. Four divergence time points from TimeTree (http://timetree.org.cn, accessed date: 23 February 2023) were used to calibrate the divergence times: (a) *Callorhinchus milii* and *Latimeria chalumnae* (421.5–461.6 MYA), (b) *L. chalumnae* and *Lepisosteus oculatus* (416.4–422.2 MYA), (c) *L. oculatus* and *Anguilla Anguilla* (372.4–383.4 MYA) and (d) *Xiphophorus maculatus* and *Oryzias latipes* (122.3–138.4 MYA).

Based on the core-orthologous gene sets, we used MCscanX v1.5.2 [31] to define syntenic blocks between *P. chinensis* and *H. transpacificus*, *P. chinensis* and *E. Lucius.* The core-orthologous gene sets were identified using Blast (v2.0.14) with an E-value threshold of 1 × 10^−5^ (at least 20 syntenic genes allowed), and visualized using NGenomeSyn v1.41 (https://github.com/hewm2008/NGenomeSyn, accessed date: 23 February 2023). We used CAFE v2.1 [32] to calculate the overall *P-value* of each branch and node with the Viterbi method.

### 2.6. Transcriptome under Air Exposure Stress

During the harvesting of *P. chinensis*, hypoxia frequently arises when they are removed from water, leading to significant mortality due to oxygen deprivation. Based on a preliminary experiment, exposure to air for more than 10 min resulted in massive mortality of the larvae. We designed an experiment to simulate the hypoxia process with five groups (the larvae fish re-entered the water after 10 min out of the water, and they either died immediately (DIC), died 15 min later (DIF), died half an hour later (DHH), died an hour later (DOH), or remained alive an hour later (UOH)). Subsequently, the clean reads were aligned onto the CDS of *P. chinensis* using Bowtie2 v 2.4.1 [33]. Gene expression levels were measured using a software package named RSEM [34]. The fragments per kilobase of exon per million mapped reads (FPKM) [35] method was used to calculate the expression level. In total, 17,299 genes were detected in at least one of the samples (FPKM ≥ 1). Additionally, a total of 1921 DEGs were detected via pairwise comparison with R package DESeq2 [36]. Subsequently, trend analysis was performed using the OmicShare tools (https://www.omicshare.com/tools, accessed date: 23 February 2023).

## 3. Results

### 3.1. Chromosome-Scale Genome Assembly

**Figure 2:**
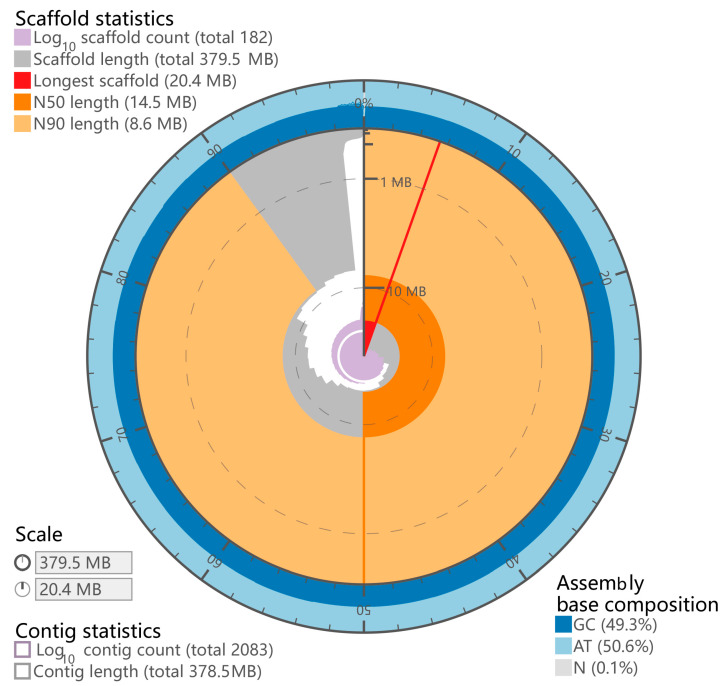
Genome characteristics of *P. chinensis*.

**Figure 3:**
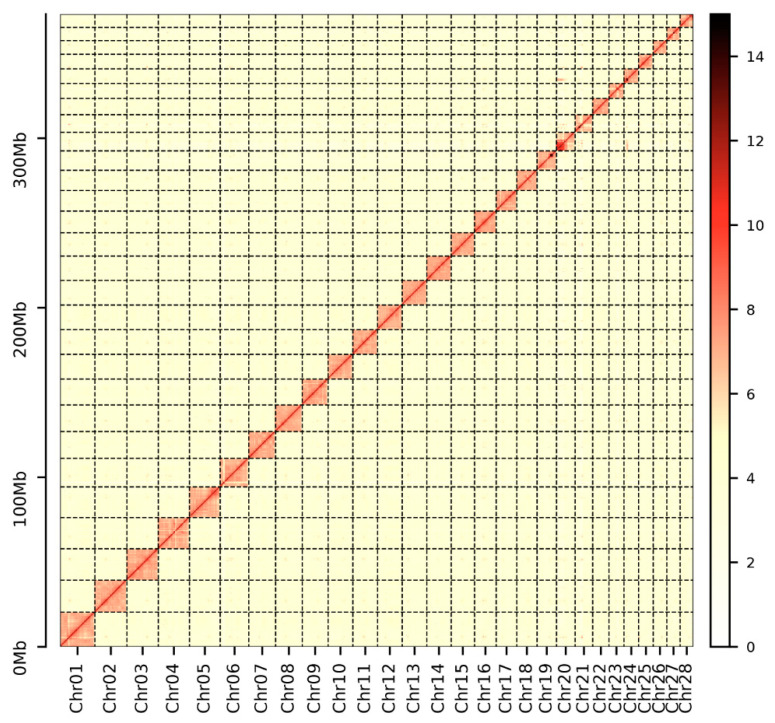
Genome-wide Hi-C heatmap of *P. chinensis*.

After sequencing, we obtained 45 Gb short-insert-size data for the *P. chinensis* genome survey (Table S1). The genome size of *P. chinensis* was estimated to be ~392.80 Mb with 0.68% heterozygosity (Figure S1 and Table S2). A total size of 378.20 Mb genome assembly with a contig N50 of 0.53 Mb was acquired (Table S3). Notably, the size of contig N50 was 5-fold longer than *P. chinensis* [3] and 25-fold longer than *P. hyalocranius* [2] (Table S3). Finally, we constructed a better *P. chinensis* genome assembly with 379.47 Mb genome size with a scaffold N50 size of 14.52 Mb, and 98.35% of the assembly sequences were assigned to 28 pseudochromosomes (Figure 2 and Figure 3 and Table S4). 

### 3.2. Repetitive Sequences

In total, we identified 35.35% (134.14 Mb) repeat sequences of the *P. chinensis* genome assembly, of which the highest proportion was DNA transposons (16.42%), followed by long interspersed nuclear elements (LINEs, 11.73%), and long terminal repeats (LTRs, 8.17%) (Tables S5 and S6, Figure S2).

### 3.3. Genome Annotation

In total, we have identified 21,074 protein-coding genes with an average gene length of 8017 bp and average coding sequence (CDS) length of 1695 bp. The average exon number per gene was 10 with an average exon length of 175 bp, and an average intron length of 727 bp (Table S7). To evaluate the credibility of gene annotation, we calculated the overlap ratio between the *P. chinensis* gene models and the prediction results from de novo, homolog-based, and RNA-seq prediction. 99.66% of *P. chinensis* gene models were supported by at least one piece of evidence at the level of greater than 80% CDS overlap ratio (Table S8). Notably, the length of gene models had a similar distribution trend at the genes, CDS, exons and introns level, compared to *D. rerio*, *O. latipes*, *P. hyalocranius* and *S. salar* (Figure S3). The Benchmarking Universal Single-Copy Orthologs (BUSCO) test, referencing the 3640 actinopterygii protein set, identified 88.10% of gene sets as complete, 83.40% of the complete actinopterygii (odb10) genes were found (Table S9). 

In addition, we aligned 95.7% of coding proteins into seven functional databases, including the non-redundant (NR) protein database of NCBI (95.25%), SwissProt [37] (87.64%), Kyoto Encyclopedia of Genes and Genomes (KEGG) [38] (86.15%), KOG (76.36%), Translation of European Molecular Biology Laboratory (Trembl) (95.33%), InterPro (91.59%), and Gene Ontology (GO) databases (68.66%) (Table S10 and Figure S4).

### 3.4. Phylogenetic and Gene Family Analysis

**Figure 4:**
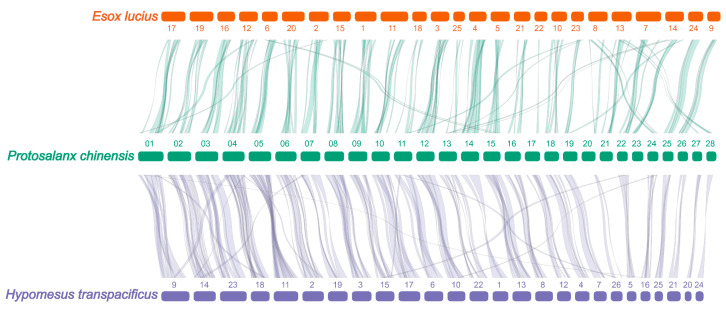
Genome synteny between *P. chinensis* and *H. transpacificus*, *P. chinensis*, and *E. lucius*.

Finally, 357,384 protein-coding genes were clustered into 29,939 gene families in 15 Osteichthyes and 1 Chondrichthyes (Figure S5). Of these, 347,307 (97.18%) genes were identified as 19,862 orthologous groups (Table S11). Our analysis indicated that *P. chinensis* and *H. transpacificus* diverged about 115.5 MYA, and *P. chinensis* and *Esox lucius* diverged about 256.2 MYA (Figure S6). *P. chinensis* and *H. transpacificus* had a highly similar synteny to *P. chinensis* vs. *E. Lucius* (Figure 4 and Table S12).

**Figure 5:**
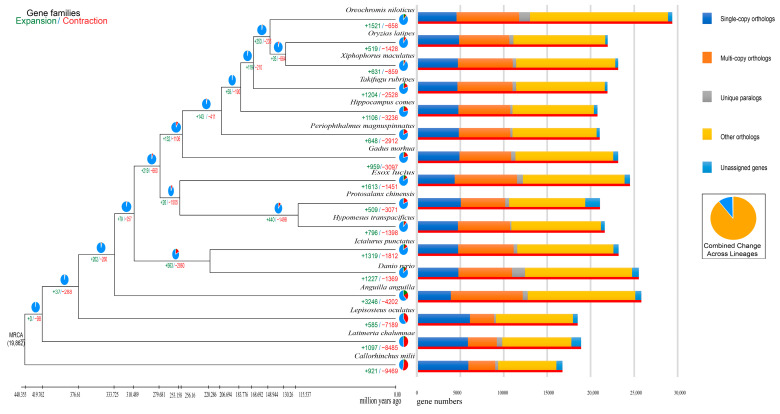
Number of expanded and contracted gene families in *P. chinensis.*

Compared to the last common ancestor, *P. chinensis* genome had a total of 509 expanded gene families and 3701 contracted gene families (Figure 5). Of these, 103 expanded gene families (including 1373 genes) and 86 contracted gene families (including 81 genes) were calculated to be markedly changed with a *p*-value less than 0.01 (Figure 5). Through KEGG enrichment analysis, we observed that the significant expansion of gene families was mainly enriched into pathways like the pentose phosphate pathway, galactose metabolism, fructose and mannose metabolism, and so on (Figure S7 and Table S13), while the significant extraction gene families were mainly clustered into pathways including ascorbate and aldarate metabolism (Figure S8, Tables S14 and S15).

### 3.5. Gene Expression under Air Exposure Stress

**Figure 6:**
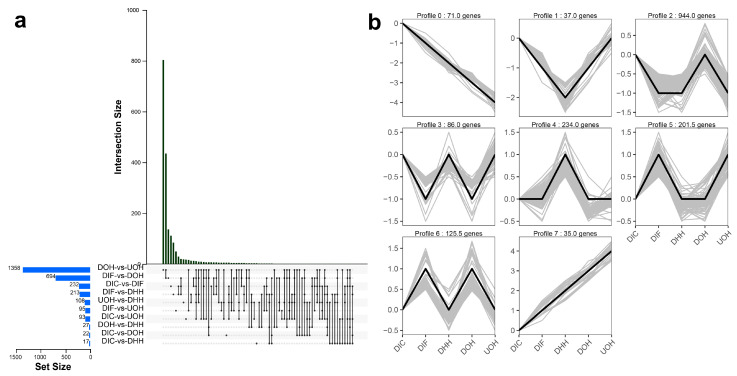
DGEs analysis. (**a**) UpSetR plots depicting the number of unique and shared DEGs. (**b**) Trend analysis of DEGs expression profiles.

Three biological replicates were included for each group, which yielded approximately 96.4 Gb clean reads in total (Table S16). In total, 17,299 genes were detected in at least one of the samples (FPKM ≥ 1). Furthermore, 1921 DEGs were detected. We observed that the expression pattern of 35 DEGs had an upward trend, whereas the expression profile of 71 DEGs demonstrated a downward trend (Figure 6). Upward trending DEGs were significantly enriched in circadian rhythm, glycosaminoglycan biosynthesis—keratan sulfate, lysosome and phagosome. Downward trending DEGs were significantly enriched in circadian rhythm—fly, circadian rhythm, arginine and proline metabolism, dopaminergic synapse, IL-17 signaling pathway, and oxytocin signaling pathway (Tables S17 and S18). In the circadian rhythm pathway, *bhlh* gene and *cry1* gene were consistently upregulated, while *clock* gene, *arntl* gene, and *rorb* gene were consistently downregulated.

## 4. Discussion

The high-quality fish genome serves as a transformative key into the intricate world of aquatic life, revealing the evolutionary history, environmental adaptations, and potential applications for aquaculture [39,40]. The *P. chinensis* has attracted the attention of researchers due to its unique biological properties and economic value [2,3]. With the development of genomics technology, researchers have been able to study the genome of *P. chinensis*. In terms of genome quality, our genome exceeds that of previous genomes. The use of PacBio HIFI sequencing for library construction provides higher accuracy and longer read-continuity compared to previous PacBio CLR and WGS sequencing [2,3]. As a result, the genome assembly generated from HiFi data has improved contiguity, as indicated by an increased contig N50. The size of contig N50 was 5-fold longer than *P. chinensis* [3] and 25-fold longer than *P. hyalocranius* [2] (Tables S3 and S4). The size of scaffold N50 was 2.8-fold longer than *P. chinensis* [3] and 12-fold longer than *P. hyalocranius* [2] (Tables S3 and S4). And we assembled 28 pseudochromosomes, with 98.35% of the assembled sequences attributed to 28 chromosomes. We believe that our study can provide more detailed basic information for the study of the germplasm resources of *P. chinensis*.

By constructing a phylogenetic tree of 16 fish species, including *P. chinensis*, our results equally support previous studies that Osmeriformes (*P. chinensis*) acts as a sister order to Esociformes (*E. lucius*) [3]. This revelation underscores the close evolutionary affinity between these two orders, elucidating the shared ancestry and evolutionary trajectories that have shaped their distinct yet interconnected genetic landscapes. Delving deeper into the annals of evolutionary history within the Osmeriformes order, we directed our attention towards an intriguing comparison of divergence times between *Hypomesus* (*H. transpacificus*) and *Protosalanx* (*P. chinensis*). The time of divergence between the two genera is estimated to be about 115.5 MYA, i.e., the Cretaceous period.

As with the genomes of most fish, the repetitive elements of *P. chinensis* form a large part of the genome. Our comprehensive analysis of the *P. chinensis* genome assembly unveiled a rich tapestry of repeat sequences, amounting to a total of 35.35% (134.14 Mb) of the genome. Within this repetitive landscape, we observed the dominance of DNA transposons, constituting the largest proportion at 16.42%. Following closely behind were long interspersed nuclear elements (LINEs) at 11.73% and long terminal repeats (LTRs) at 8.17% (Tables S5 and S6, Figure S2). These findings shed light on the intricate composition and dynamics of repetitive elements within the *P. chinensis* genome, paving the way for further investigations into their functional significance and evolutionary implications.

Previous studies on the feeding habits of *P. chinensis* have shown that there is a shift in feeding habits from phytoplankton to carnivorous after the juvenile stage of *P. chinensis* [41]. Analysis of gene families showed that 103 gene families including 1373 genes had been expanded. Interestingly, these genes are significantly enriched in metabolic pathways such as galactose metabolism, fructose and mannose metabolism, biosynthesis of amino acids, carbon metabolism, and metabolic pathways. Although the expansion or contraction of gene families may be the result of random and natural selection and difficult to prove [42], the potential association between the expansion of these genes and the feeding habits of *P. chinensis* provides an important reference for future studies.

Air exposure stress is a useful method for studying the physiological responses of aquatic economic animals under environmental stress. In commerce, fish and shrimps encounter environmental stresses of hypoxia during capture, loading and transport. And studying the physiological characteristics and performance of aquatic economic animals under hypoxic conditions can help improve their survival rates and reduce losses [43,44]. In evolutionary terms, air exposure stress also helps to explore potential mechanisms of aquatic to terrestrial evolution [45]. Due to the difficult availability and high mortality rate of adult *P. chinensis*, we selected available larvae at early developmental stages as experimental subjects to analyse physiological trends through their transcriptional expression. 

In the enrichment pathway of the upregulation trend, lysosomal and phagosome responses are important predictors of environmental stress in aquatic animals [46]. Lysosome and phagosome pathway related genes indicated that the organism of *P. chinensis* exhibited a continuous environmental stress response under the air exposure stress. In addition, IL-17 signaling showed a downward trend. The IL-17 signaling pathway is thought to be important for the maintenance of health under physiological stress [47]. Studies have shown that IL-17 plays a role in the resistance of fish to bacterial infection and is involved in the immune response. Differential expression of IL-17 family genes in skin tissues occurred in turbot after infection with *Vibrio anguillarum* [48]. The persistent decline of IL-17-related genes during air exposure stress in *P. chinensis* may indicate that air exposure stress affects the immune function of *P. chinensis* fingerlings to some extent.

Interestingly, pathway enrichment analysis showed that these two clusters of genes were significantly enriched in circadian rhythm pathways. Circadian rhythm-related genes showed both significant and consistent upregulation and consistent downregulation. Some studies have shown that the circadian rhythm is associated with a stress response in fish [49]. Significant circadian rhythm changes in heart rate in rainbow trout have been observed after stress from transport, etc. [50]. A study on spiny ginseng suggests that circadian rhythms are involved in the response to heat, hypoxia, and thermo-hypoxic stress through DNA methylation [51]. Although the specific function and role of circadian rhythm-related genes during stress in *P. chinensis* could not be clarified in this study, the specific mechanism of action deserves further investigation.

Genes associated with circadian rhythms are affected in fish following environmental stresses such as temperature, light, and hypoxia [52,53,54]. A hypoxia exposure study in *Phoxinus lagowskii* showed that sustained hypoxia exposure resulted in significant upregulation of *cry1b* gene. Under diel-cycling hypoxia exposure, the expression of three of the eight clock genes was increased, including *per1a*, *clocka*, and *cry1b* [53]. *cry1a* and *cry1b* expression is increased by heat shock in zebrafish ZEM-2S cells under a light–dark cycle [52]. Similarly, cold shock leads to dysregulation of the expression of genes such as zebrafish circadian rhythms, phototransduction and the IL-17 signing pathway and causes inflammation [54]. In this study, we showed that the expression of *clock* genes was changed in the larvae of *P. chinensis* under air exposure stress, indicating that hypoxic stress induced dysregulation of physiological rhythms in *P. chinensis*.

Future studies on the genome of *P. chinensis* should enhance the assembly of the “Telomere to Telomere” (T2T) genome by increasing long-read ONT sequencing [55]. It is also essential to validate the assembled genome’s accuracy using methods like PCR or Sanger sequencing. An in-depth exploration of comparative and functional genomics can uncover more biological significance. By incorporating these improvements, the study’s methodological robustness and reliability can be improved, resulting in more detailed and persuasive findings.

These genomic datasets aimed at unraveling the ecological dynamics and adaptive mechanisms of *P. chinensis*. Furthermore, they provided invaluable insights into the species’ ability to cope with air exposure stress, shedding light on the molecular underpinnings of its resilience. Beyond these immediate implications, our findings have far-reaching implications for the conservation and preservation of this remarkable species, elevating our understanding of its unique biological makeup and enabling more effective conservation strategies to safeguard its future.

## 5. Conclusions

In summary, a better *P. chinensis* genome assembly with 379.47 Mb genome size was reported with a scaffold N50 size of 14.52 Mb, and 98.35% of the assembly sequences was assigned to 28 pseudochromosomes. The expansion of gene families related to galactose metabolism, fructose and mannose metabolism, biosynthesis of amino acids, and carbon metabolism pathways in the genome of *P. chinensis* provides novel insights. Based on the intricate patterns of gene expression, our findings unveil the remarkable physiological activities and molecular responses exhibited by *P. chinensis* in hypoxic conditions. The circadian rhythm pathway is likely to be critical in the process of air exposure stress in *P. chinensis*, and its role cannot be ignored. These insights serve as a crucial stepping-stone towards unraveling the intricate mechanisms governing the species’ survival and adaptation strategies, further expanding our understanding of the complex interplay between genetics, physiology, and environmental stressors.

## Data Availability

The raw genome sequencing data for *P. chinensis* were deposited in the NCBI Sequence Read Archive (SRA) database under Accession the BioProjectID PRJNA915822. The genome assembly, genome annotation, coding sequences, protein sequences, repeat annotation and functional annotation files were deposited in Figshare: https://doi.org/10.6084/m9.figshare.22144694.v1 (accessed date: 23 February 2023).

## Supplementary Materials

The following supporting information can be downloaded at: https://www.mdpi.com/article/10.3390/biology12091266/s1, Figure S1: 21-mers analysis for estimating the genome size of *P. chinensis*; Figure S2: Divergence distribution of repetitive elements in *P. chinensis* genome; Figure S3: Distribution of gene, coding sequence, exon, and intron lengths, and exon number in *P. chinensis* and other four genomes; Figure S4: Gene function annotation results in the five databases of NR, InterPro, KEGG, SwissProt and KOG statistics Venn diagram; Figure S5: Phylogenetic tree of 16 species based on maximum-likelihood using 2152 single-copy orthologs; Figure S6: Estimation of divergence times of 16 species; Figure S7: Functional enrichment results of expansion gene families in *P. chinensis* genome, Terms with *p* < 0.01 was selected; Figure S8: Functional enrichment results of extraction gene families in *P. chinensis* genome, Terms with *p* < 0.01 was selected; Table S1: Sequencing data used for the genome *P. chinensis* assembly; Table S2: The information of *P. chinensis* genome survey analysis; Table S3: The statistics of length and number for the de novo assembled *Protosalanx* genomes; Table S4: Statistics of chromosomal length of *P. chinensis* genome; Table S5: Repetitive sequences in *P. chinensis* genome; Table S6: Transposable elements in *P. chinensis* genome; Table S7: Gene predictions in *P. chinensis* genome; Table S8: The evidence supporting the gene models of *P. chinese* genome; Table S9: BUSCO analysis result of *P. chinensis* genome; Table S10: Functional annotations of *P. chinensis* genes; Table S11: Gene family clustered; Table S12: The statics of Syntenic Blocks; Table S13: Top 20 pathway resulted from KEGG. KEGG enrichment of the markable expanded gene family in *P. chinensis* genome; Table S14: Top 20 pathway resulted from KEGG. KEGG enrichment of the markable extracted gene family in *P. chinensis* genome; Table S15: Data for analysis in this study; Table S16: RNA map ratio; Table S17: KEGG Pathway enrichment analysis for upward trend DEGs; Table S18: Pathway enrichment analysis for downward trend DEGs.
