# Supplementary material for: Chromosome-Level Genome Assembly of Protosalanx chinensis and Response to Air Exposure Stress
Source: Biology (Basel). 2023 Sep 21;12(9):1266. doi: 10.3390/biology12091266 (PMC10525151; doi:10.3390/biology12091266)
Supplement: Supplementary file 1 [file biology-12-01266-s001.zip › biology-2459085-supplementary.pdf]

Chromosome-level genome assembly of Protosalanx chinensis and response to air exposure stress

Supplementary:

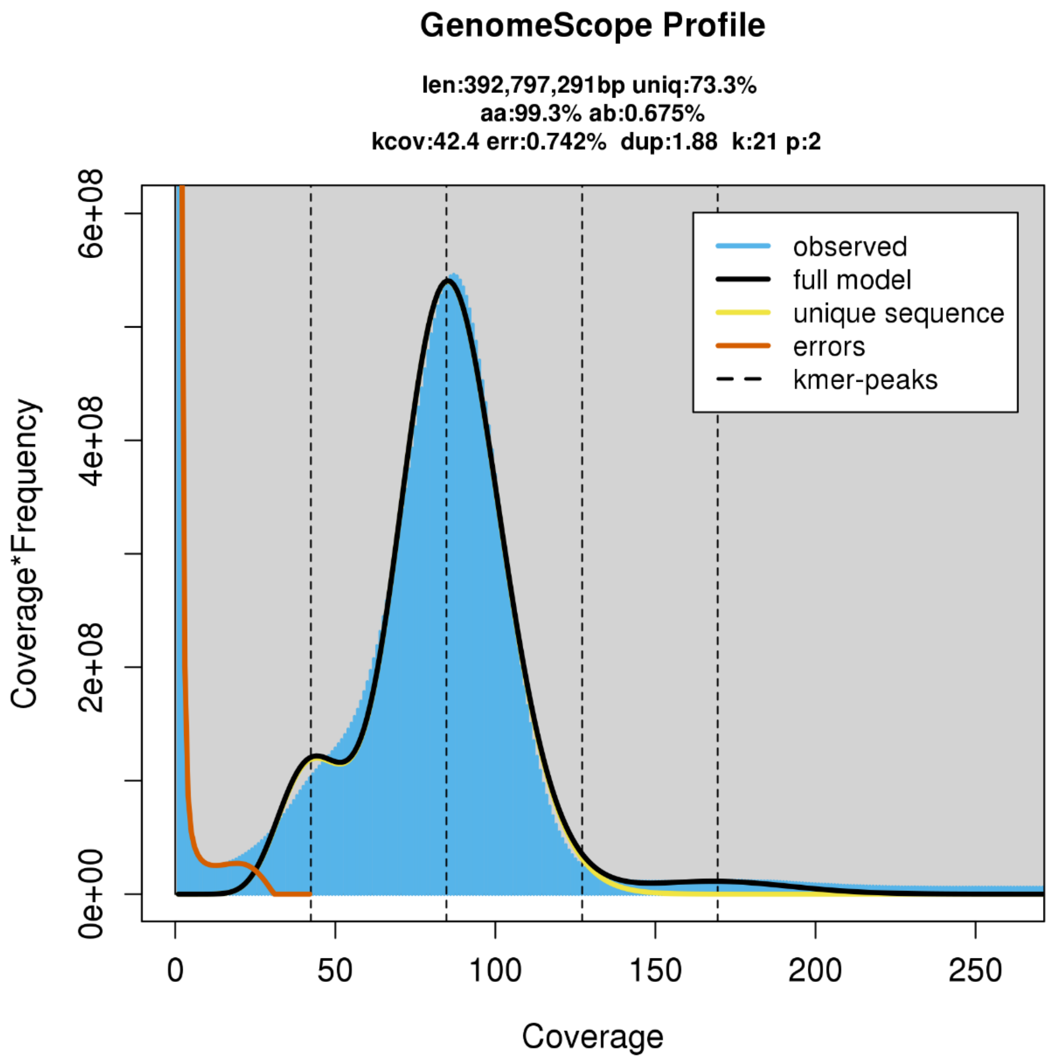

Figure S1. 21-mers analysis for estimating the genome size of *P. chinensis.*

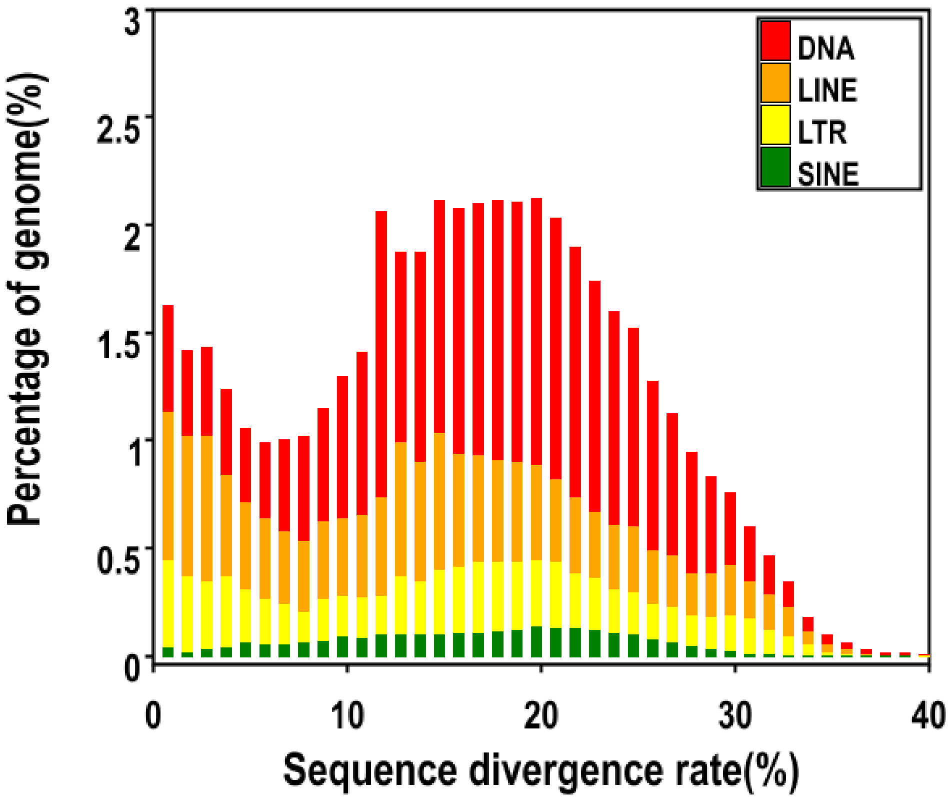

Figure S2. Divergence distribution of repetitive elements in *P. chinensis* genome.

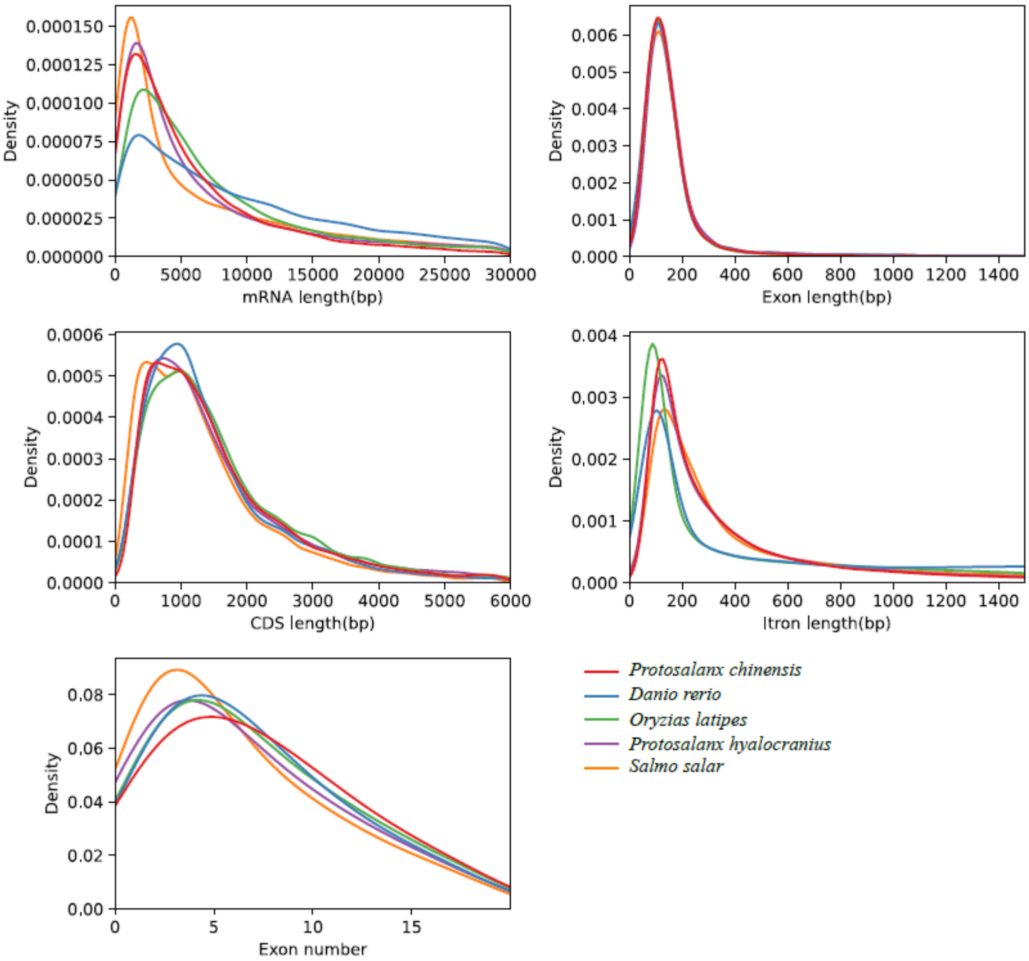

Figure S3. Distribution of gene, coding sequence, exon, and intron lengths, and exon number in *P. chinensis* and other four genomes.

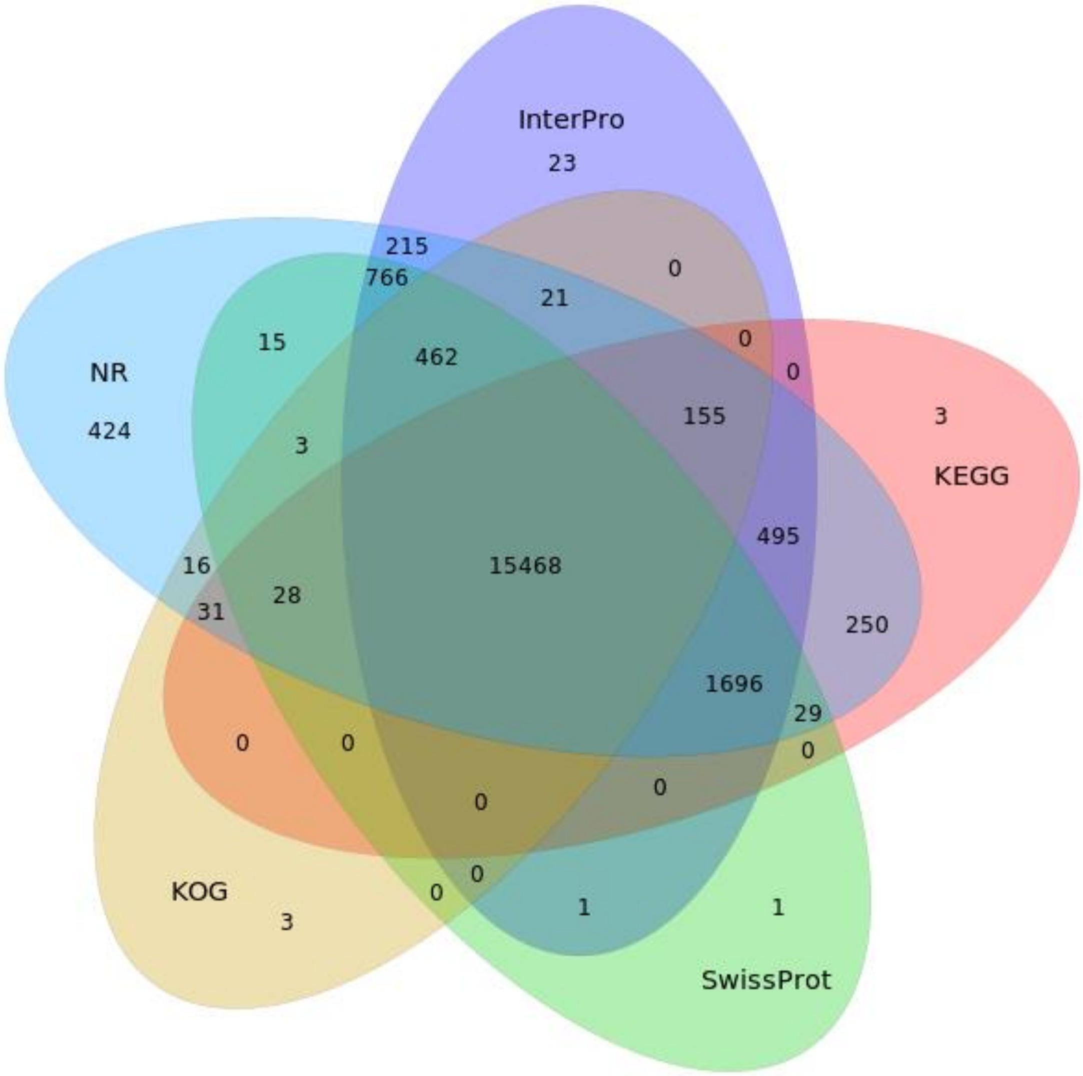

Figure S4. Gene function annotation results in the five databases of NR, InterPro, KEGG, SwissProt and KOG statistics Venn diagram.

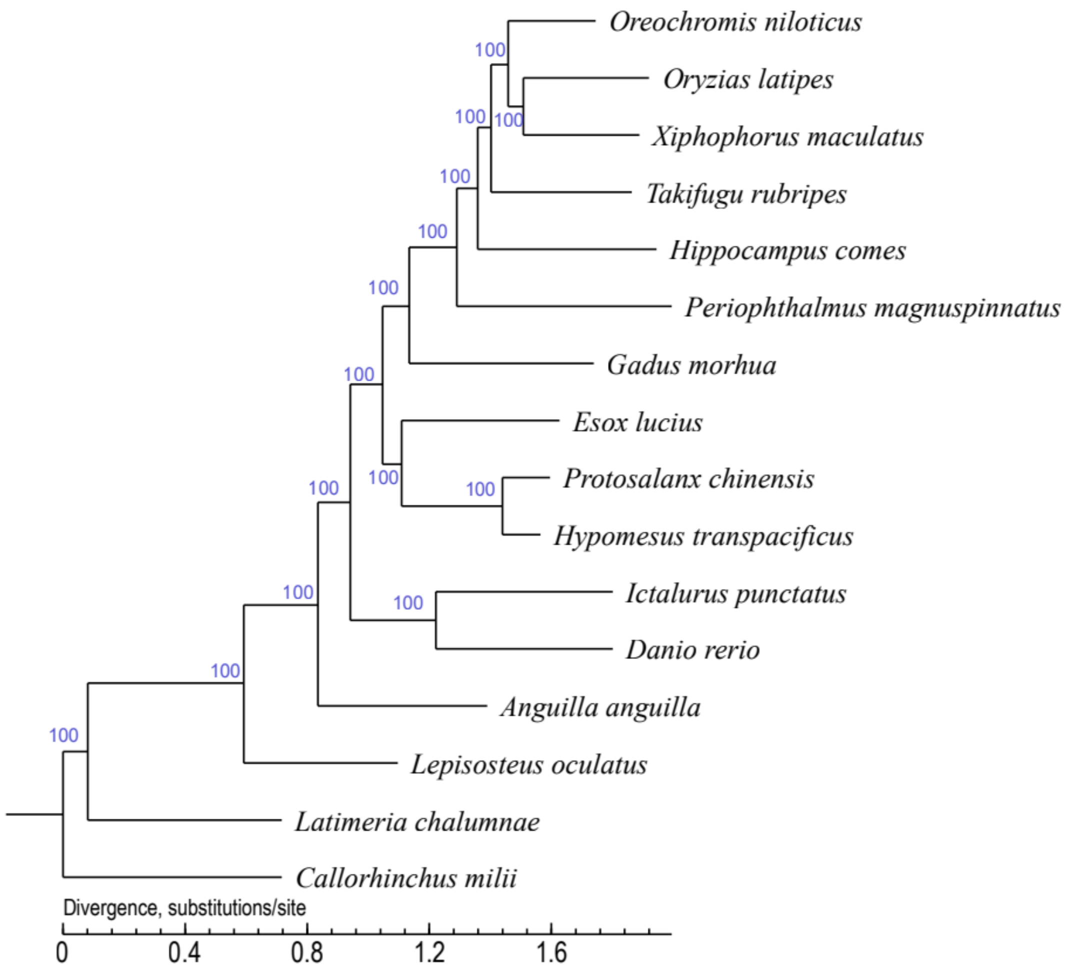

Figure S5. Phylogenetic tree of 16 species based on maximum-likelihood using 2 152 single-copy orthologs.

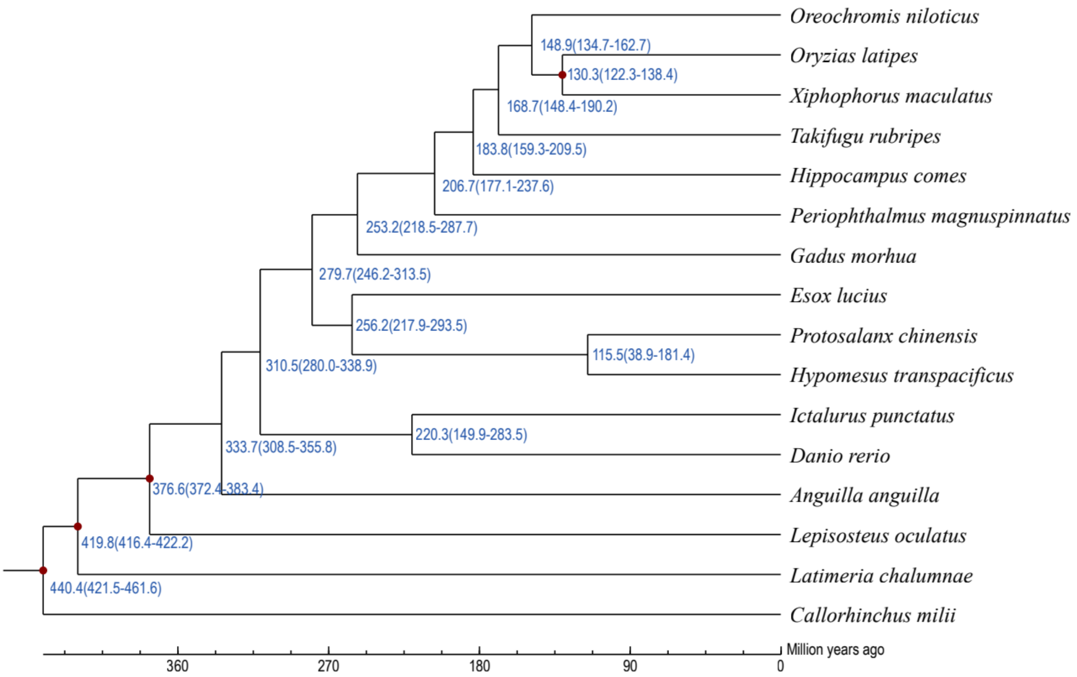

Figure S6. Estimation of divergence times of 16 species.

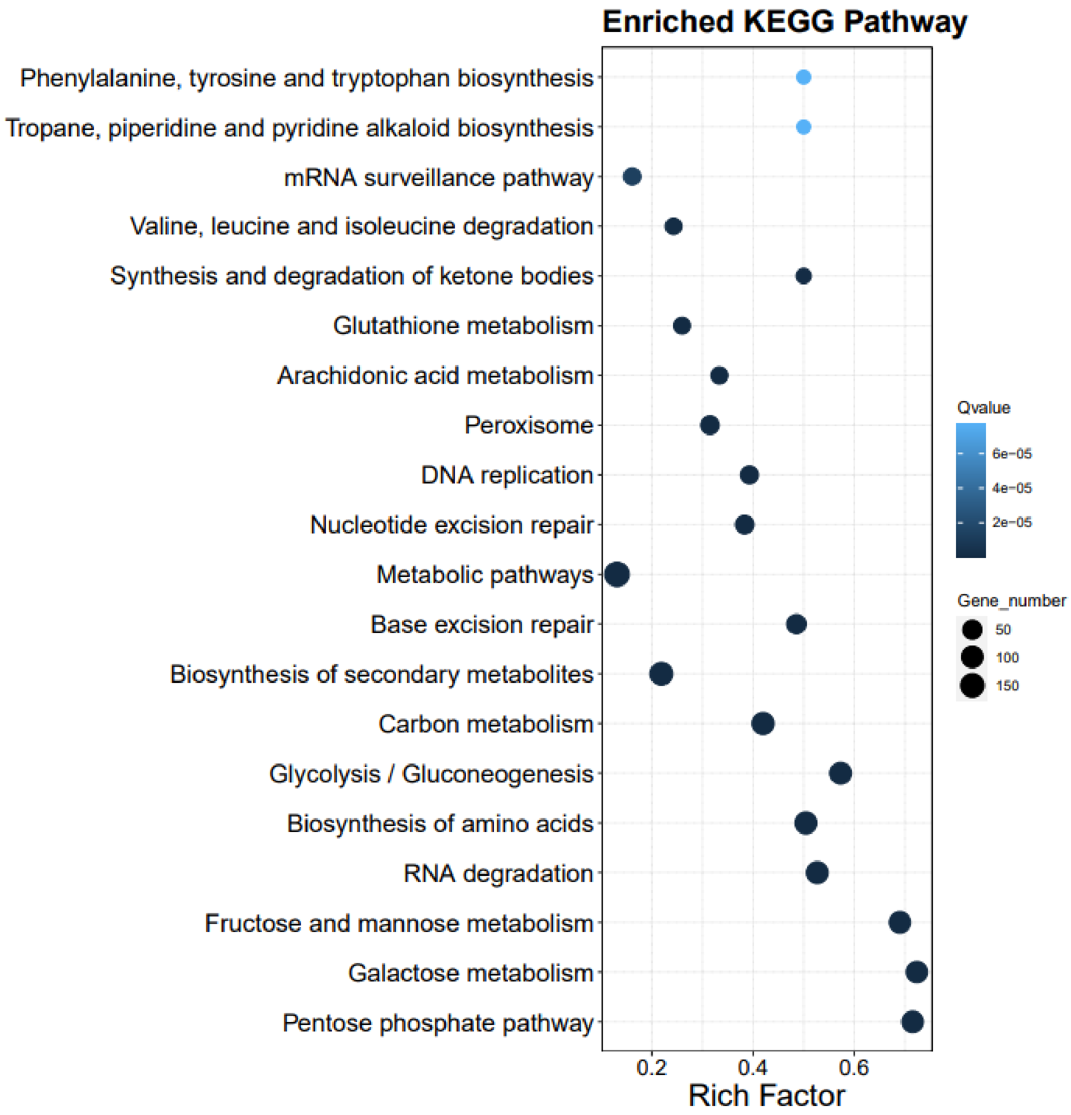

Figure S7. Functional enrichment results of expansion gene families in *P. chinensis* genome, Terms with $P < 0.01$ was selected.

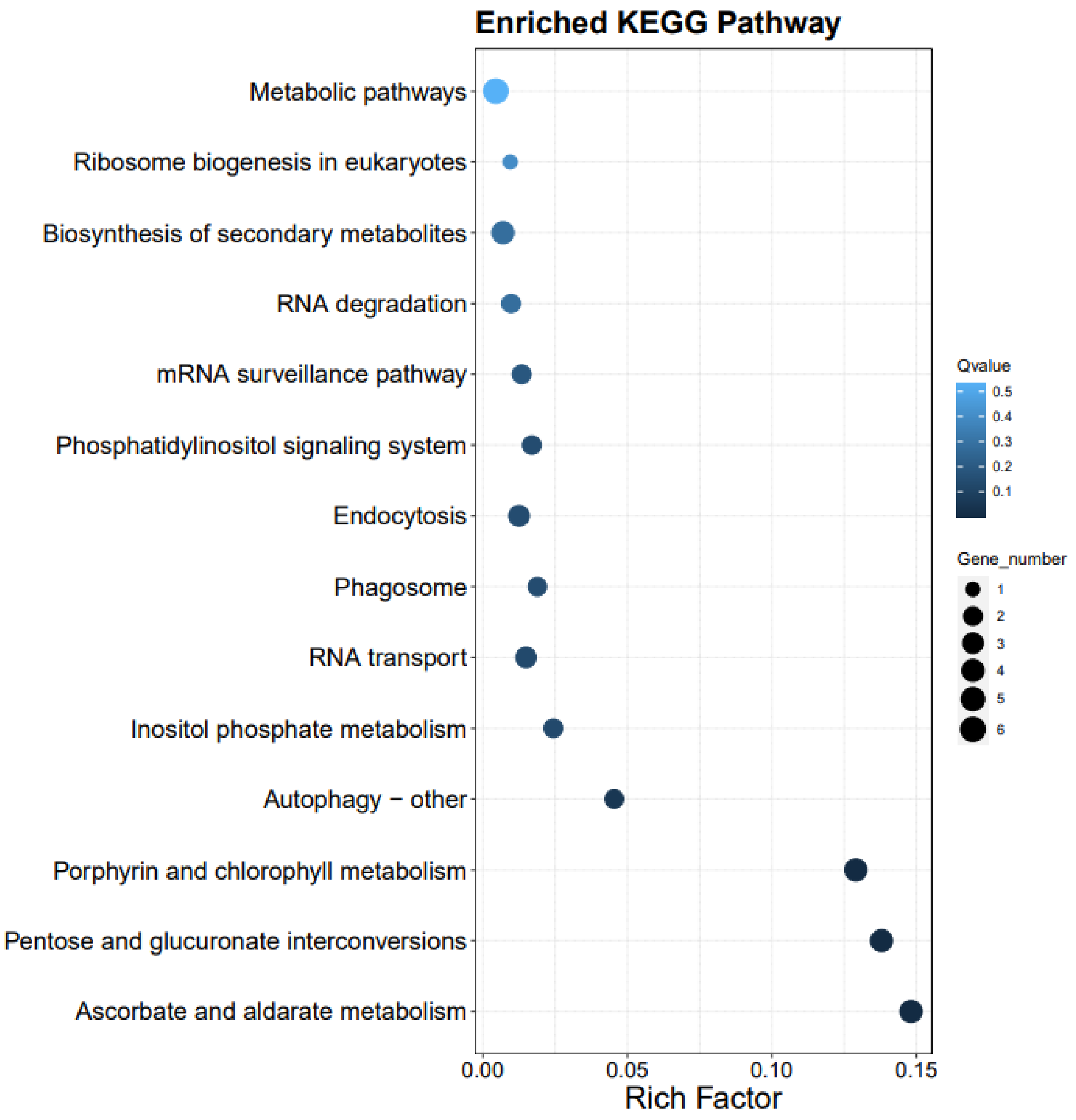

Figure S8. Functional enrichment results of extraction gene families in *P. chinensis* genome, Terms with *P*<0.01 was selected.

Table S1 Sequencing data used for the genome *P. chinensis* assembly.

Type	Sample	Platform	Bases / Gb	Reads Count	Max length / bp	Mean length / bp	N50 / bp
CCS	muscle	PacBio Sequel II (Hifi)	25	1,350,737	37,330	18,271	18,205
Hi-C	muscle	DNBSEQ	107	356,700,000	150	150	150
WGS	muscle	DNBSEQ	45	150,066,667	150	150	150
RNAseq	muscle	DNBSEQ	96.4	321,333,333	150	150	150

Note: Clean data.

Table S2 The information of *P. chinensis* genome survey analysis.

Kmer	Kmer number	Used Base /Gb	Genome Size/Mb	Heter Rate/%	Repeat rate/%	Error Rate	Depth/X
21	38,889,443,073	46.55	392.80	0.68	26.70	0.74	119.36

Table S3 The statistics of length and number for the de novo assembled Protosalanx genomes.

	Protosalanx chinensis *				*Protosalanx chinensis* [#1]				*Protosalanx hyalocranius* [#2]			
	**scaffold**		**contig**		**scaffold**		**contig**		**scaffold**		**contig**	
	**Length (Mb)**	**number**	**Length (Mb)**	**number**	**Length (Mb)**	**number**	**Length (Mb)**	**number**	**Length (Mb)**	**number**	**Length (Mb)**	**number**
**Total length**	379.47		378.20		466.69		444.87		536.56		414.85	
**Max length**	20.42		6.23		44.19		2.14		5.40		0.37	
**number>=2000bp**		182		2,083		618		11,196		681		19,755
**N50**	14.52	12	0.53	138	5.189	23	0.10	876	1.16	126	0.02	5,716
**N60**	14.37	14	0.32	227	4.03	33	0.06	1,443	0.89	179	0.01	8,488
**N70**	12.14	17	0.20	377	2.57	48	0.03	2,424	0.72	246	0.009	12,448
**N80**	10.38	21	0.12	629	1.63	71	0.02	4,260	0.53	334	0.005	18,481
**N90**	8.60	25	0.06	1,090	0.79	110	0.01	7,942	0.40	451	0.003	28,932

Note: "*" indicated this study. #1 indicated data from NCBI (GCA_010882115.1). #2 indicated data from GigaDB.

Table S4 Statistics of chromosomal length of *P. chinensis* genome

ID	length / bp	N content / %	GC content / %	gap number
Chr01	20,420,276	0.12	45.23	51
Chr02	18,896,921	0.19	45.77	72
Chr03	18,499,308	0.21	45.71	76
Chr04	18,387,637	0.21	45.66	77
Chr05	18,113,639	0.22	46.00	78
Chr06	16,854,175	0.25	45.69	83
Chr07	15,801,697	0.20	45.77	62
Chr08	15,704,969	0.18	46.32	56
Chr09	15,250,643	0.29	46.18	88
Chr10	14,596,046	0.22	46.46	65
Chr11	14,559,807	0.25	46.00	73
Chr12	14,524,669	0.20	45.76	58
Chr13	14,516,431	0.28	46.71	81
Chr14	14,368,868	0.22	45.92	62
Chr15	13,763,165	0.25	46.24	69
Chr16	12,769,249	0.29	45.96	74
Chr17	12,140,554	0.29	46.49	70
Chr18	11,942,187	0.26	46.49	63
Chr19	11,457,059	0.30	46.54	69
Chr20	10,973,078	0.27	45.97	60
Chr21	10,382,235	0.35	46.38	72
Chr22	9,567,638	0.34	46.47	65
Chr23	8,865,516	0.34	45.35	61
Chr24	8,622,590	0.44	46.79	76
Chr25	8,599,918	0.33	46.09	56
Chr26	8,044,071	0.38	45.89	61
Chr27	7,821,795	0.45	46.56	70
Chr28	7,775,152	0.34	46.92	53
UnChr	7,684	0.00	49.89	154*

Table S5 Repetitive sequences in *P. chinensis* genome.

Type	Repeat Length(bp)	% of genome
**Trf**	49,646,207	13.08
**Repeatmasker**	55,900,805	14.73
**Proteinmask**	13,034,864	3.44
**De novo**	106,663,727	28.11
**Total**	134,135,271	35.35

Table S6 Transposable elements in *P. chinensis* genome.

Type	Repbase Length	TEs (Bp)	TE %	protiens in	De genome	novo Length	Combined (Bp)	TEs %
DNA	37,198,648	9.80	2,152,173	0.57	45,396,534	11.96	62,315,343	16.42
LINE	14,962,624	3.94	8,259,127	2.18	38,685,479	10.19	44,528,459	11.73
SINE	2,236,495	0.59	0	0.00	7,458,771	1.97	8,955,619	2.36
LTR	9,678,765	2.55	2,630,607	0.69	24,310,203	6.41	31,005,092	8.17
Other	4,367	0.0012	207	0.0001	0	0.00	4,574	0.0012
Unknown	0	0.00	0	0.00	2,854,747	0.75	2,854,747	0.75
Total	55,900,805	14.73	13,034,864	3.44	101,482,109	26.74	110,718,260	29.18

Table S7 Gene predictions in *P. chinensis* genome.

	Gene set	Number	Average gene length(bp)	Average CDS length(bp)	Average exon per gene	Average exon length(bp)	Average intron length(bp)
De novo	***Augustus***	32,797	5,303	1,436	7	205	645
	Snap	53,282	6,210	1,178	6	185	935
**Homolog**	***D. rerio***	19,501	7,014	1,604	9	174	658
	O. latipes	20,409	6,813	1,605	9	185	678
	P. hyalocranius	18,912	7,561	1,566	8	195	854
	S. salar	25,318	6,357	1,512	8	184	672
**Transcript**		55,612	8,985	1,296	7	186	1,286
**Final**		21,074	8,017	1,695	10	175	727

Table S8 The evidence supporting the gene models of *P. chinese* genome

	>=30% overlap		>=50% overlap		>=80% overlap	
	**No.**	**Ratio (%)**	**No.**	**Ratio (%)**	**No.**	**Ratio (%)**
**R(single)**	1,101	5.22	1,239	5.88	1,773	8.41
**H(single)**	6	0.03	14	0.07	187	0.89
**H(more)**	5	0.02	22	0.1	249	1.18
**P(single)**	6	0.03	47	0.22	376	1.78
**P(more)**	70	0.33	229	1.09	580	2.75
**HR**	629	2.98	915	4.34	2,346	11.13
**PR**	1,577	7.48	1,517	7.2	1,279	6.07
**PH**	2,127	10.09	2,677	12.7	3,406	16.16
**PHR**	15,553	73.8	14,414	68.4	10,807	51.28
**Total**	21,074	100	21,074	100	21,003	99.66

Note: "P" refers to evidences from *de novo* prediction; "H" refers to evidences from homolog prediction; "R" refers to evidences from transcriptomic prediction; "PH" refers to evidences both from *de novo* prediction and homolog prediction; "PR" refers to evidences both from transcriptomic prediction and *de novo* prediction; "HR" refers to evidences both from transcriptomic prediction and homolog prediction; "PHR" refers to evidences from transcriptomic prediction, homolog prediction and *de novo* prediction ; "single" refers to one type of evidence; "more" refers to more than one evidence. ">=30% overlap" refers to 30% of the query sequences are aligned onto target sequences; ">=50% overlap" refers to 50% of the query sequences are aligned onto target sequences; ">=80% overlap" refers to 80% of the query sequences are aligned onto target sequences;

Table S9 BUSCO analysis result of *P. chinensis* genome

Type	Genome		Protein	
	**Number**	**Percentage (%)**	**Number**	**Percentage (%)**
**Complete BUSCOs**	3,208	88.10	3,035	83.40
**Complete and single-copy BUSCOs**	3,109	85.40	2,959	81.30
**Complete and duplicated BUSCOs**	99	2.70	76	2.10
**Fragmented BUSCOs**	71	2.00	94	2.60
**Missing BUSCOs**	361	9.90	511	14.00
**Total BUSCO groups searched**	3,640	100.00	3,640	100.00

Note: actinopterygii_odb10 database

Table S10 Functional annotations of *P. chinensis* genes.

	Total	Nr	Swissprot	KEGG	KOG	TrEMBL	Interpro	GO	Overall
**Number**	21,074	20,074	18,469	18,155	16,187	20,090	19,302	14,470	20,140
**Percentage/%**	100	95.25	87.64	86.15	76.81	95.33	91.59	68.66	95.57

Table S11 Gene family clustered.

Species	Number of genes	Number of genes in orthogroups	Number of unassigned genes	Number of species-specific orthogroups	Number of genes in species-specific orthogroups
Anguilla anguilla	25,827	25,127	700	91	595
Callorhinchus milii	16,762	16,058	704	91	348
Danio rerio	25,534	24,762	772	172	1,466
Esox lucius	24,524	23,887	637	101	665
Gadus morhua	23,169	22,581	588	104	507
Hippocampus comes	20,772	20,352	420	74	249
Hypomesus transpacificus	21,614	21,176	438	37	156
Ictalurus punctatus	23,205	22,602	603	103	417
Latimeria chalumnae	18,904	17,763	1,141	108	616
Lepisosteus oculatus	18,514	17,946	568	50	224
Oryzias latipes	21,972	21,667	305	74	476
Oreochromis niloticus	29,385	28,884	501	220	1,257
Protosalanx chinensis	21,074	19,376	1,698	81	436
Periophthalmus magnuspinnatus	21,032	20,679	353	52	241
Takifugu rubripes	21,946	21,614	332	88	417
Xiphophorus maculatus	23,150	22,833	317	54	387

Table S12 The statics of Syntenic Blocks.

SpecieA vs SpecieB	Of Synteni c Blocks	Average Syntenic Gene Pairs Per Block	Of Syntenic Gene Pairs	Mean Block Length of SpecieA	Mean Block Length of SpecieB
P. chinensis vs E. lucius	187	36	6,725	1,306,983	2,701,623
P. chinensis vs P. transpacificus	194	57	11,012	1,774,393	1,936,891

Note: at least 20 genes required to call a collinear block between inter-species.

Table S13 Top 20 pathway resulted from KEGG. KEGG enrichment of the markable expanded gene family in *P. chinensis* genome.

Pathway	Gene_number	Backgroud_gene_number	RichFactor	Qvalue
Pentose phosphate pathway	98	137	0.72	1.71E-89
Galactose metabolism	97	134	0.72	1.71E-89
Fructose and mannose metabolism	98	142	0.69	2.92E-87
RNA degradation	108	205	0.53	3.71E-78
Biosynthesis of amino acids	111	220	0.50	9.10E-78
Glycolysis / Gluconeogenesis	98	171	0.57	8.65E-76
Carbon metabolism	115	274	0.42	1.58E-69
Biosynthesis of secondary metabolites	126	576	0.22	5.43E-40
Base excision repair	51	105	0.49	1.22E-34
Metabolic pathways	176	1344	0.13	8.21E-26
Nucleotide excision repair	41	107	0.38	4.82E-23
DNA replication	33	84	0.39	4.35E-19
Peroxisome	40	127	0.31	6.07E-19
Arachidonic acid metabolism	22	66	0.33	2.65E-11
Glutathione metabolism	20	77	0.26	3.17E-08
Synthesis and degradation of ketone bodies	9	18	0.50	7.77E-07
Valine, leucine and isoleucine degradation	17	70	0.24	1.08E-06
mRNA surveillance pathway	24	149	0.16	1.36E-05
Tropane, piperidine and pyridine alkaloid biosynthesis	6	12	0.50	7.74E-05
Phenylalanine, tyrosine and tryptophan biosynthesis	6	12	0.50	7.74E-05

Table S14 Top 20 pathway resulted from KEGG. KEGG enrichment of the markable extracted gene family in *P. chinensis* genome.

Pathway	Gene_ number	Backgroud_ gene_number	RichFactor	Qvalue
Ascorbate and aldarate metabolism	4	27	0.15	4.46E-05
Pentose and glucuronate interconversions	4	29	0.14	4.46E-05
Porphyrin and chlorophyll metabolism	4	31	0.13	4.46E-05
Autophagy - other	2	44	0.05	5.50E-02
Inositol phosphate metabolism	2	82	0.02	1.34E-01
RNA transport	3	201	0.01	1.34E-01
Phagosome	2	106	0.02	1.45E-01
Endocytosis	3	240	0.01	1.45E-01
Phosphatidylinositol signaling system	2	118	0.02	1.45E-01
mRNA surveillance pathway	2	149	0.01	1.92E-01
RNA degradation	2	205	0.01	2.82E-01
Biosynthesis of secondary metabolites	4	576	0.01	2.82E-01
Ribosome biogenesis in eukaryotes	1	106	0.01	4.00E-01
Metabolic pathways	6	1344	0.00	5.35E-01

Table S15 Data for analysis in this study

Species	Data Accession	Data Source
*Protosalanx chinensis**	NA	This Study
Protosalanx chinensis	GCA_010882115.1	NCBI
Protosalanx hyalocranius	https://ftp.cngb.org/pub/gigadb/pub/ 10.5524/100001_101000/100262/	GigaDB
Anguilla anguilla	GCF_013347855.1	NCBI
Callorhinchus milii	GCF_018977255.1	NCBI
Danio rerio	GCF_000002035.6	NCBI
Esox lucius	GCF_011004845.1	NCBI
Gadus morhua	GCF_902167405.1	NCBI
Hippocampus comes	GCF_001891065.1	NCBI
Hypomesus transpacificus	GCF_021917145.1	NCBI
Ictalurus punctatus	GCF_001660625.2	NCBI
Latimeria chalumnae	GCF_000225785.1	NCBI
Lepisosteus oculatus	GCF_000242695.1	NCBI
Oryzias latipes	GCF_002234675.1	NCBI
Oreochromis niloticus	GCF_001858045.2	NCBI
Takifugu rubripes	GCF_901000725.	NCBI
Xiphophorus maculatus	GCF_002775205.1	NCBI

Table S16 RNA map ratio

Sample	Clean read /Mb	q20/%	q30/%	Total mapping Gene ratio/%	Uniquely mapping gene ratio/%	Total mapping genome ratio/%	Uniquely mapping genome ratio/%
DIC1	64.92	97.47	89.99	59.41	46.92	87.63	80.95
DIC2	65.29	97.53	90.14	59.26	46.39	88	81.12
DIC3	65.44	97.59	90.39	62.17	41.49	88.87	76.38
DIF1	64.77	97.32	89.48	61.36	48.35	87.94	81.21
DIF2	62.85	97.37	89.62	62.11	46.51	88.73	80.08
DIF3	64.99	97.38	89.67	61.47	47.45	88.23	80.88
DOH1	63.59	97.54	90.07	66.08	43.07	90.3	78.91
DOH2	65.2	97.55	90.13	60.34	46.64	88.19	80.88
DOH3	66.23	97.69	90.56	66.31	40.69	90.15	76.33
UOH1	63.2	97.55	90.09	59.64	46.75	88.41	81.51
UOH2	65.55	97.69	90.57	61.15	47.79	88.24	81.34
UOH3	62.95	97.59	90.33	60.86	47.45	87.79	80.55
DHH1	63.11	97.41	89.77	63.42	44.04	89.92	79.54
DHH2	62.98	97.45	89.86	59.43	45.42	88.06	79.8
DHH3	62.88	97.67	90.54	60.02	47.52	87.44	80.99

Table S17 KEGG Pathway enrichment analysis for upward trend DEGs.

Pathway	Profile7 (26)	All-gene (18155)	Pvalue	Pathway ID	Level 1	Level 2	KOs
Circadian rhythm	3	56	0.00007	ko04710	Organismal Systems	Environmental adaptation	K03729+K02295
Glycosaminoglycan biosynthesis - keratan sulfate	1	19	0.02688	ko00533	Metabolism	Glycan biosynthesis and metabolism	K04745
Lysosome	2	185	0.02859	ko04142	Cellular Processes	Transport and catabolism	K05656+K01369
Phagosome	2	246	0.04802	ko04145	Cellular Processes	Transport and catabolism	K06461+K07889

Table S18 Pathway enrichment analysis for downward trend DEGs.

Pathway	Profile7 (26)	All-gene (18155)	Pvalue	Pathway ID	Level 1	Level 2	KOs
Circadian rhythm - fly	3	15	0.00002	ko04711	Organismal Systems	Environmental adaptation	K02223+K02296
Circadian rhythm	4	56	0.00003	ko04710	Organismal Systems	Environmental adaptation	K02223+K02296+K08533
Arginine and proline metabolism	3	74	0.00188	ko00330	Metabolism	Amino acid metabolism	K00286+K01581
Dopaminergic synapse	4	238	0.00791	ko04728	Organismal Systems	Nervous system	K02223+K02296+K04348
IL-17 signaling pathway	3	135	0.01010	ko04657	Organismal Systems	Immune system	K09029+K02187+K09487
Oxytocin signaling pathway	4	395	0.04149	ko04921	Organismal Systems	Endocrine system	K01242+K00907+K06625+K04348
